# Mobile Payments, Chinese Tourists, and Host Residents: Are Destination Stakeholders Prepared to Facilitate Mobile Payments?

**DOI:** 10.1007/978-3-030-65785-7_18

**Published:** 2020-11-28

**Authors:** Tania Maria Tangit, Rob Law

**Affiliations:** 1 Department for Informatics, Technical University of Munich, Garching bei München, Bayern Germany; 2 Smart Tourism Education Platform (STEP) College of Hotel and Tourism Management, Kyung Hee University, Seoul, Korea (Republic of); 3 Department of Tourism and Service Management, MODUL University Vienna, Vienna, Wien Austria; School of Hotel and Tourism Management, The Hong Kong Polytechnic University, Kowloon, Hong Kong SAR

**Keywords:** Mobile payment, Mobile payment usage behaviour, Chinese outbound tourism, Destination stakeholders, Sabah (Malaysian-Borneo)

## Abstract

Mobile payment has become ubiquitous worldwide. It is a form of digital payment activity performed on-site from a mobile device (i.e. smartphone) for the purchase of goods and services using QR or NFC (contactless and proximity card) technology. This study examines destination stakeholders’ level of engagement and involvement in facilitating mobile payments (e.g., AliPay and WeChat Pay) for Chinese tourists. Sabah (Malaysian-Borneo), a popular island destination in Southeast Asia, was selected as the study site. Using a qualitative approach, 25 tourism and hospitality industry practitioners participated in a semi-structured, in-depth interview between February to July 2020. Preliminary results revealed that industry practitioners had been actively seeking to implement mobile payment facilities since late-2017, specifically in the retail sector, as they felt the pressure in accommodating the needs of Chinese tourists. This study offers insights into how industry practitioners address local consumers’ shift in payment usage-behaviours; from traditional forms of payments (e.g., cash and credit card) to mobile payments in light of the COVID-19 pandemic.

## Mobile Payments and Chinese Tourism

The global phenomenon of mobile payment (MP) technologies is spearheaded by the Chinese (Mainland China) as they exemplify a cashless society. They consider it a norm to rely on mobile devices to make day-to-day payments even during travels [3, 4]. Prior to the COVID-19 pandemic, many destinations worldwide were overwhelmed with the influx of Chinese tourists. Considering the growth of 20% outbound tourists yearly, they contribute to the growth of tourism and the economy in many parts of the world. Destinations capitalized on Chinese spenders by implementing mobile technologies to facilitate AliPay and WeChat Pay payments. With this in mind, the Chinese tourism segment being the largest body of travellers abroad has changed the global travel landscape, specifically how destinations cater to them [5].

As MP becomes ubiquitous in many Asian countries and globally, there are drawbacks to this universal phenomenon, such as MP failures during travel attributed to merchants, network operators, service providers, and users [4]. Moreover, the barriers to implementing MP in many countries at a local or state level are their facilities, aside from installation cost, lack of trust and knowledge, as well as safety and security issues. There has been no consensus on the standard setting of MP in which its success and failures of implementation have primarily attributed to country-specific consumer’s payment culture [1]. As prerequisites of MP, the current facilities lack universality and interoperability [1, 2]. Simply put, the over-dependence on MP usage among (Chinese) travellers may pose a problem when destinations do not offer such facilities [3, 4].

Currently, MP research within the tourism and hospitality industry (e.g. hotel and restaurant setting) has made very little progress over the past several years with much focus on the Western context for MP adoption and technology acceptance. Knowing that MP is part of the Chinese social norm, it is unknown the extent of the MP culture can be observed when they travel abroad as MP facilities are not readily available at all destinations. Therefore, this study’s main objective is to understand stakeholders’ level of involvement and engagement in facilitating MP for Chinese tourists at destinations. Depending on the availability of MP facilities at destinations (country), stakeholders have generally focused on the Chinese MP model, AliPay and WeChat Pay, with less attention on local MP for host residents. Subsequently, this study examines the tourism and hospitality industry practitioners’ responses to residents’ MP needs.

## Methodology

This study is part of a large project which examines the role of MP and value co-creation efforts among destination stakeholders. This study adopted an interpretive, qualitative research approach informed by social constructionist ideologies. The data presented were collected from tourism and hospitality industry practitioners for six months (February to July 2020) in the capital city of Sabah (Malaysian-Borneo), Kota Kinabalu (KK). Sabah was selected as Chinese outbound tourists highly rated it as one of the top island destinations in 2017. Participants were selected based on convenience and snowball sampling methods from two sources (offline/online). The interview followed a semi-structured format, where participants were briefed on the research objectives, followed by their written/verbal consent to audio-recording of the interviews. The interviews were transcribed verbatim and translated to English. Interpretations were validated through member-checking and verified with a seasoned tourism expert. Thematic analysis was used to interpret data using both manual and computer-assisted analysis (NVIVO12) to identify, categorize and code patterns.

## Preliminary Findings and Discussion

In total, 25 participants agreed to participate in this study, with an average of 32-min per interview. A majority of the participants are male (68%) and married (75%), with most under the age of 31–40 years (32%) and 41–50 years (28%). Tourism and hospitality industry practitioners are identified based on industry categories: Food and Beverage (F&B), Travel, Hotel, Retail, Payments, and Wellness (Spa). Based on preliminary findings in this study, almost half of the industry practitioners fall under the category of ‘innovators’ and ‘early adopters’ (See: Diffusion of Innovation by Rogers, 1962). These stakeholders highly sought for MP technologies from late-2017 (‘Hotels’ and ‘Retail’ categories) to 2018 (‘F&B’ and ‘Wellness’ categories) and signed up with little hesitation in 2019 (‘Retail’ and ‘F&B’ categories) to cater to the Chinese tourism segment. Table 1 provides a summary of the participants in this study.

**Table 1.** Study participants’ profile and mobile payment facilitation readiness

Tourism and hospitality industry practitioners’ demographic profile (N = 25)		Mobile payment facility				*
		Yes			No	
		Local	Chinese	Both		
*Food & Beverage (F&B)*						
1.	Restaurant Manager, KK (M, 50)			/		1
2.	Durian Seller, Fruit Stall, KK (M, 35)		/			1
3.	Owner, Coffee shop, KK (M, 55)				/	1
4.	Owner, Coffee shop, KK (F, 52)				/	3
5.	Owner, Coffee shop, KK (M, 26)	/				3
*Wellness (Spa)*						
6.	Owner, Health & Wellness Centre, KK (M, 34)			/		2
*Hotel*						
7.	General Manager, 4-star Hotel, KK (M, 59)		/			3
*Retail*						
8.	Manager, Jewellery Store, KK (F, 46)		/			1
9.	Manager, Souvenir Shop, KK (F, 50)		/			1
10.	Owner, Souvenir Shop, KK (M, 58)			/		1
11.	Manager, Sports Apparel Retail Store, KK (M, 27)			/		1
12.	Manager, Souvenir Shop, KK (M, 35)			/		3
13.	Owner, Souvenir Kiosk, KK (F, 29)	/				3
*Payments*						
14.	Top Management, Payment Gateway (M, 54)	Not applicable				2
15.	Manager (Merchant Acquirer), Payment Gateway (F, 33)					2
16.	Manager (Merchant Acquirer), Malaysian eWallet (M, 46)					3
*Travel*						
17.	Manager (Accommodation), Online Travel Agency, KK (M, 29)	Not applicable				1
18.	Freelance Tour Guide – Chinese Market (M, 39)				/	1
19.	Freelance Tour Guide – Chinese Market (M, 38)		/			1
20.	Freelance Tour Guide – Chinese Market (F, 46)				/	2
21.	Freelance Tour Guide – Chinese & South Korean Market (F, 37)				/	2
22.	Freelance Tour Guide – Chinese Market (M, 48)		/			2
23.	Freelance Tour Guide – English & Chinese Market (M, 36)				/	2
24.	Tour Coordinator, Inbound/Outbound Operator, KK (F, 29)		/			2
25.	Tour Operator (Product Owner), KK (M, 50)	/				2

*Interaction via Offline/Face-to-face (1 and 3) and Online/Phone (2): 1 = mid-Feb to mid-Mar 2020; 2 = mid-Mar to mid-May 2020 (MCO: Movement Control Order in Malaysia); 3 = mid-May to mid-July 2020

In the service industry, it is common for hotels within the 4- to 5-star range in Sabah to have MP facilities for Chinese guests, but rarely for local guests. However, a spa owner (#6) revealed that having both local and Chinese MP systems ease sales consolidation for all forms of payments (cash, credit cards, and MP) across all branch outlets. Most establishments in the retail industry (n = 6) accepts Chinese MP with three retailers (#10, #11 and #12) accepting local MP as well. For example, participant #12 relayed that their main customers are domestic tourists; hence, the need to provide local MP facility on top of accepting Chinese MP.

In the ‘Travel’ category (n = 9), one inbound/outbound operator (#24) accepts Chinese MP apart from accepting major credit cards, bank transfers, and cash transactions. However, one tour operator (#25) does not accept Chinese MP as the current business deals with local tour agents (intermediaries) who pay in cash or bank transfer. Chinese guests usually pay for tour packages via OTAs (e.g., CTrip), which accept Chinese MP (#17). There are fewer Chinese FIT guests who book directly with the tour operator, and when they do, the transactions are either in cash or credit card (#25). There are two Chinese-speaking, freelance tour guides (#19 and #22) who accept Chinese MP via P2P transfers as they own a Chinese eWallet (e.g., WeChat Pay) linked to their Chinese banking account (i.e., both have worked in Mainland China prior). All tour guides agreed that their Chinese guests are more inclined to spend at retail stores with Chinese MP. However, tour guides are usually prepared with cash (Malaysian Ringgit) when their Chinese guests request to exchange local currencies.

This study also found that F&B merchants such as participants #3 and #4 have yet to adopt MP facilities. Their reservations are due implementation cost and merchants’ lack of know-how in consolidating payments via MP systems. For example, a local durian seller (#2) shares one WeChat account with a few other sellers (within the same vicinity). Moreover, participants #3 and #4 differentiated food businesses (medium-large businesses with branch outlets) that rely heavily on workers would most probably use MP facilities as compared to small-medium, family-run businesses. Participants in the ‘Payments’ industry (#14, #15 and #16) further corroborated that most merchants are reluctant to enrol for MP (local, Chinese or both) because they do not want additional payment methods. Their reluctance is due to keeping their businesses as uncomplicated and straightforward as possible by accepting ‘cash only’. Nonetheless, the impact of the COVID-19 pandemic led participant #5 to apply for local MP facility in order to cope with the growing need for food deliveries during the MCO period.

Although (lack of) ‘trust’ was cited in past literature, this study shows that industry practitioners were eager and ready to facilitate payments for Chinese tourists and, of late, adopt local MP due to the COVID-19 pandemic. It is worth noting that utilizing MP facilities have been equated as being hygienic (i.e., safe and free from germs) for destination hosts. For example, two participants (#5 and #13, 25–30 year-old) implemented local MP facilities (i.e., QR code display) during Malaysia’s MCO period to facilitate payments for its customers (e.g., food take-away and in-app food delivery services). Additionally, they aspire to capture the future domestic tourist segment.

One of the key findings of this study is that local natives are less likely to adopt MP facilities as they perceive that such technologies are not safe and secure (e.g. data privacy). They feel additional costs will be incurred for setting the facility, in addition to not fully understanding how payments are received at their end. For local merchants who adopted Chinese MP, they believe it limits the need for local staff to communicate with Chinese tourists due to cultural differences and language barrier. Furthermore, MP adoption among stakeholders varies, considering the industry characteristics and their dependence on the Chinese tourism segment. For example, the retail industry provides MP solutions to enhance consumers’ shopping experience by preserving the sense of convenience and familiarity, making tourism accessible to the Chinese tourist segment. This preliminary finding is similar to Yang [5], where merchants at host destinations are compelled to accommodate Chinese tourists. Further analysis is required to link residents’ MP usage behaviour with the growing Chinese outbound tourism.

## Conclusion and Limitations of the Study

Globally, the COVID-19 pandemic has contributed to the accelerated shift in payment preference; from physical payments (i.e., cash bills) to digital (mobile) payments. As a result, MP technologies will play an integral role to see a wider adoption of both suppliers/merchants and buyers/consumers worldwide. In the foreseeable future, destination stakeholders alongside with governmental efforts would require to not only bridge the digital (mobile) gap through ICT infrastructures for its residents and visitors but to also concentrate on MP security and verification features (e.g. blockchain technologies). As payment methods have an impact on consumer behaviour, future studies may delve into consumers’ (tourists) personal and socio-cultural values related to MP usage behaviours, particularly on product/service consumptions, as they have social, marketing, and legal implications. On limitations, this study was conducted at the beginning of 2020, where the social/physical distancing practices may have contributed to participants’ reluctance in this study. Moreover, it cannot be denied that insider-bias may be a limitation in selecting tourism industry practitioners as the primary researcher of this study is a Sabah native.

## References

[1] Carr M (2009) Framework for mobile payment systems in India. In: Head M, Li EY (eds) Mobile and ubiquitous commerce: advanced e-business methods. Hershey & New York, IGI Global, pp 237–254

[2] Karnouskos S, Fokus F (2004). Mobile payment: a journey through existing procedures and standardization initiatives. IEEE Commun Surv Tutor.

[3] Law R, Sun S, Schuckert M, Buhalis D (2018) An exploratory study of the dependence on mobile payment among Chinese travelers. In: Stangl B, Pesonen J (eds) Information and communication technologies in tourism 2018. Springer, Cham

[4] Sun S, Law R, Zhong L.: Mobile payment failure during travel. J China Tour Res, 1–17 (2019)

[5] Yang ICM, French, JA, Lee LMQ, Shrestha, KM (2020) An institutional isomorphism perspective of tourism impact. Ann Tour Res, 102921

